# Exploring the Potential of Anthocyanins for Repairing Photoaged Skin: A Comprehensive Review

**DOI:** 10.3390/foods13213506

**Published:** 2024-11-01

**Authors:** Xinmiao Guo, Linlin He, Jiaqiang Sun, Hua Ye, Cuiyuan Yin, Weiping Zhang, Hao Han, Wengang Jin

**Affiliations:** 1 School of Biological Science and Engineering, Shaanxi University of Technology, Hanzhong 723001, China; 18717215515@163.com (X.G.); sunjiaqiang24@163.com (J.S.); yh18716289047@163.com (H.Y.); 15691611707@163.com (C.Y.); kjc_zwp@163.com (W.Z.); hanhao183@snut.edu.cn (H.H.); 2 Shaanxi Province Key Laboratory of Bio-Resources, Shaanxi University of Technology, Hanzhong 723001, China; 3 Qinba Mountain Area Collaborative Innovation Center of Bioresources Comprehensive Development, Shaanxi University of Technology, Hanzhong 723001, China; 4 Qinba State Key Laboratory of Biological Resources and Ecological Environment (Incubation), Shaanxi University of Technology, Hanzhong 723001, China; 5 Shaanxi Black Organic Food Engineering Center, Shaanxi University of Technology, Hanzhong 723001, China

**Keywords:** ultraviolet, skin photoaging, anthocyanins, occurrence, functional foods

## Abstract

Long-term exposure to ultraviolet (UV) rays can result in skin photoaging, which is primarily characterized by dryness, roughness, pigmentation, and a loss of elasticity. However, the clinical drugs commonly employed to treat photoaged skin often induce adverse effects on the skin. Anthocyanins (ACNs) are water-soluble pigments occurring abundantly in various flowers, fruits, vegetables, and grains and exhibiting a range of biological activities. Studies have demonstrated that ACNs contribute to the repair of photoaged skin due to their diverse biological characteristics and minimal side effects. Evidence suggests that the stability of ACNs can be enhanced through encapsulation or combination with other substances to improve their bioavailability and permeability, ultimately augmenting their efficacy in repairing photoaged skin. A growing body of research utilizing cell lines, animal models, and clinical studies has produced compelling data demonstrating that ACNs mitigate skin photoaging by reducing oxidative stress, alleviating the inflammatory response, improving collagen synthesis, alleviating DNA damage, and inhibiting pigmentation. This review introduces sources of ACNs while systematically summarizing their application forms as well as mechanisms for repairing photoaged skin. Additionally, it explores the potential role of ACNs in developing functional foods. These findings may provide valuable insight into using ACNs as promising candidates for developing functional products aimed at repairing photoaged skin.

## 1. Introduction

Skin aging is a complicated biological process in which the structure and function of the skin gradually deteriorate over time [1]. It can be categorized into two primary types: endogenous skin aging, which is a natural skin aging process influenced by age-related factors, such as hormones, and metabolic factors; and exogenous skin aging, which is primarily driven by ultraviolet (UV) radiation, commonly referred to as skin photoaging [2,3]. While ultraviolet radiation (UVR) can reduce surface microorganisms and enhance skin metabolism to some extent, it also poses a risk of skin photoaging. Specifically, long-wave UVA (315–400 nm) penetrates the dermis and may even reach the subcutaneous layer, leading to alterations in dermal connective tissue. Medium-wave UVB (280–315 nm) affects the epidermis and upper dermis, resulting in changes to the structural integrity of the skin [4,5,6]. Fortunately for our skin’s health, short-wave UVC (200–290 nm) is absorbed by the ozone layer before reaching the Earth’s surface [7]. The histological characteristics of photoaged skin are characterized by decreased fibroblasts, increased inflammatory cells, and the degradation of collagen and elastin [8]. Clinically observable signs of photoaging encompass dryness, peeling, thickening of the skin, loss of suppleness, and pigmentation irregularities [9].

At present, the pharmacological management of photoaging primarily involves vitamin A and its derivatives, which are regarded as the gold standard for both prevention and treatment [10]. The topical application of retinoic acid has been demonstrated to enhance levels of type I collagen in skin affected by photoaging; however, prolonged use may result in adverse reactions such as dermatitis, erythema, desquamation, and other side effects [11]. In addition to pharmacological options, cosmetic procedures such as facial injections of botulinum toxin and soft tissue fillers are commonly employed. Nevertheless, these interventions carry inherent risks including abrasions, pain, and the potential impairment of eyelid function [12]. With ongoing advancements in modern medicine, there is an increasing recognition of low-risk natural ingredients that can assist in repairing UV-induced skin damage. These include polysaccharides [13], polyphenols [14], peptides [15], and anthocyanins (ACNs) [16]. Among these compounds, ACNs—water-soluble flavonoids found in various foods—play a crucial role in functional nutrition and overall human health [17].

**Figure 1:**
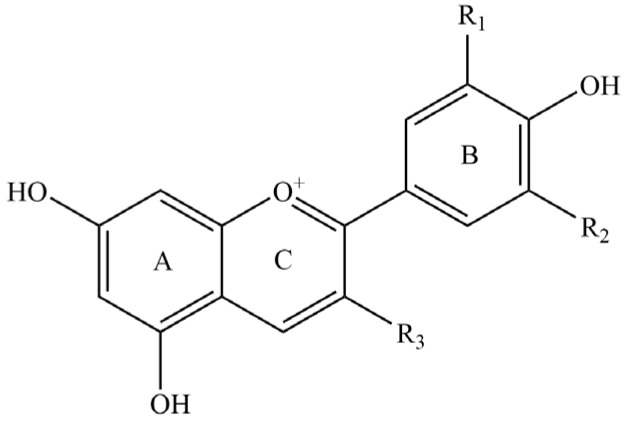
Chemical structure of anthocyanins (ACNs). Anthocyanins, ACNs; R_1_, (H, OH, OCH_3_); R_2_, (H, OH, OCH_3_); R_3_, (O-Glucoside including β-D-Glucoside, β-D-Galactose, α-L-Rhamnose, α-L-Arabinose, Sophorose, Rutinose, etc.).

The aglycone of ACNs consists of anthocyanidins characterized by a distinctive C6-C3-C6 skeleton, with a 2-phenylbenzopyran cation serving as the core framework, as illustrated in Figure 1 [18]. There are six primary types of anthocyanidins found in nature: malvidin, delphinidin, pelargonidin, peonidin, cyanidin, and petunidin. Anthocyanidins are categorized based on the presence and quantity of hydroxyl or methyl oxygen substitutions at the R_1_/R_2_ carbon atom positions on the B ring [19]. They are known to be less stable and readily bind to sugars such as rhamnose, galactose, arabinose, glucose, fructose, and other sugars through glycosidic bonds to create more stable ACNs [20]. Studies have demonstrated that ACNs possess potential health benefits for humans, including anti-cancer properties [21], anti-diabetes effects [22], anti-obesity effects [23], a reduction in blood lipids [24], and vascular protection [25]. In addition, ACNs provide beneficial biological functions for skin health such as antioxidant activity [26], anti-inflammatory effects [27], antibacterial properties [28], and protection against photodamage [29].

In recent years, the depletion of the ozone layer has resulted in an increase in the UVR intensity on the Earth’s surface, presenting a significant environmental challenge that heightens the risk of skin diseases such as photoaged skin and even skin cancer. Consequently, addressing skin photoaging induced by UVR is a critical concern for individuals. Traditional clinical treatments for photoaged skin often come with adverse side effects. In contrast, ACNs exhibit natural, environmentally friendly attributes with minimal associated risks, demonstrating promising potential in mitigating skin photoaging. This review aims to introduce sources of ACNs, systematically summarize their various application forms, elucidate mechanisms through which ACNs repair photoaged skin, and explore their potential as functional foods. These insights may enhance our understanding of skin photoaging while providing new perspectives on developing ACNs as functional foods aimed at repairing photoaged skin.

## 2. Materials and Methods

We utilized databases such as Web of Science, Google Scholar, and PubMed to search for research articles and review articles on the mechanisms underlying UVR-induced skin photoaging and its treatment involving ACNs. Keywords included photoaging, natural aging, ultraviolet radiation, skin, and anthocyanins.

## 3. Sources of ACNs

**Figure 2:**
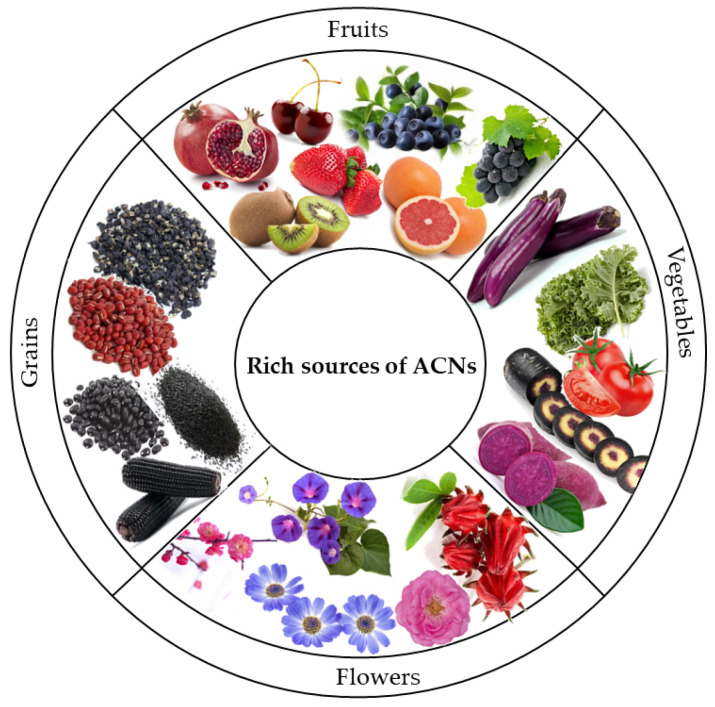
Sources of anthocyanins (ACNs).

ACNs are among the chromogenic substances responsible for producing vibrant red, black, blue, or purple colors observed in fruits, vegetables, grains, and flowers, as illustrated in Figure 2. The ACNs content varies across different food types; in fruits, it ranges from 2 to 66 mg/100 g. In berries specifically, this concentration can reach up to 611 mg/100 g. Vegetables typically contain between 3 and 75 mg/100 g of fresh weight while grains have approximately 1.3 to 103.78 mg/100 g [30,31]. This indicates that berries serve as a primary source of ACNs, highlighting their appeal not only in terms of taste and color but also underscoring their importance within a healthy diet due to their abundant ACNs content. Additionally, various grains such as purple rice, black buckwheat, purple corn, black beans, and black sorghum contribute significantly to the dietary intake levels of ACNs among individuals [32]. Moreover, numerous flower varieties exhibiting purple, blue, and red hues, including chrysanthemum, herbaceous peony, and butterfly pea, also contain these valuable compounds [33,34,35].

Different fruits, grains, and vegetables contain varying types and concentrations of ACNs monomers. Cyanidin-3-*O*-glucoside (C3G) is one of the most prevalent ACNs found in most fruits, while malvidin glycosides and their derivatives are characteristic ACNs present in red grapes [36,37]. C3G serves as the primary component of ACNs extracted from Karonda fruit [38]. Additionally, the predominant ACNs in blackberry and strawberry are C3G and geranium-3-*O*-glucoside, respectively. Furthermore, research has revealed that 32% of cyanidin-3-*O*-sophoroside and 31% of C3G constitute the main ACNs in red raspberry; notably, C3G accounts for 82% of the total ACNs content in red currant extract [39]. Black rice extract is abundant in C3G, paeoniflorin-3-glucoside, cyanidin-3,5-glucoside, and other active ingredients; within this context, C3G emerges as the principal component of black rice’s ACNs profile [40]. Moreover, peonidin along with cyanidin-3-sophoroside-5-glucoside and their acylated derivatives represent the main ACNs identified across ten varieties of purple sweet potatoes sourced from different regions in China [41]. These vibrant foods serve as rich sources of ACNs and dietary fiber, which confer various health benefits to humans.

## 4. Beneficial Effects of ACNs in Repairing Photoaged Skin

**Figure 3:**
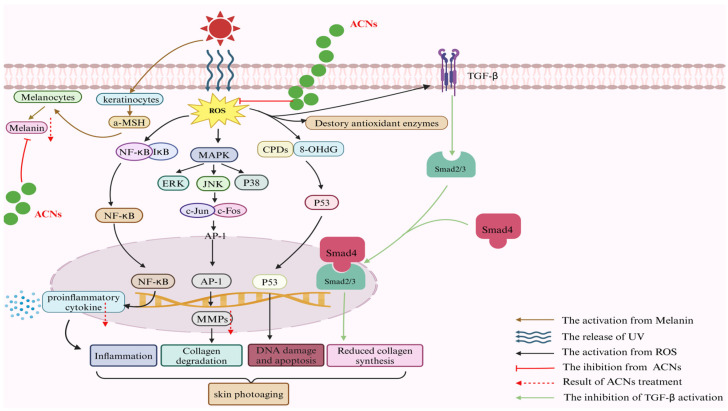
The protective mechanisms of anthocyanins (ACNs) on UVR-induced skin damage. ACNs, anthocyanins; UV, ultraviolet; UVR, ultraviolet radiation; ROS, reactive oxygen species; MAPK, mitogen-activated protein kinases; ERK, extracellular regulated protein kinases; JNK, Jun N-terminal kinase; AP-1, activator protein-1; MMPs, matrix metalloproteinases; Smad, small mothers against decapentaplegic; NF-κB, nuclear factor-kappa B; IκB, inhibitor of NF-κB; α-MSH, α-melanocyte-stimulating hormone; CPDs, cyclobutane pyrimidine dimers; 8-OHdG, 8-oxo-7, 8-dihydro-20-deoxyguanosine.

ACNs and foods rich in ACNs are beneficial to human health, as extensively reported in the literature. Their primary functions include reducing oxidative stress, inhibiting inflammatory responses, inhibiting matrix metalloproteinases (MMPs) production, reducing DNA damage, and preventing skin pigmentation issues. Collectively, these properties contribute to enhancing the extracellular matrix (ECM) structure of the skin and improving its overall appearance. Moreover, various in vitro and in vivo studies have confirmed that ACNs possess anti-photoaging effects while underscoring their beneficial impact on repairing photoaged skin (Figure 3).

### 4.1. ACNs Reduce Oxidative Stress

UVR interacts with natural photosensitizers present in the skin, leading to the excessive production of ROS, which disrupts the delicate balance of the body’s antioxidant defense system and results in damage to cells and tissues [42]. Furthermore, ROS can initiate lipid peroxidation processes that harm cell membranes [43]. In addition, ROS may hinder the activity of crucial antioxidant enzymes such as catalase (CAT), glutathione peroxidase (GSH-PX), and superoxide dismutase (SOD). Furthermore, C3G inhibits glutathione (GSH) consumption and malondialdehyde (MDA) production [44]. GSH depletion reduces overall antioxidant capacity within the body, rendering it more susceptible to ROS attacks, leading to an accumulation of ROS over time, thereby intensifying oxidative stress [45].

Oxidative stress is an important factor contributing to skin aging. However, it is believed that ACNs effectively protect against these detrimental effects due to their high content of active hydroxyl groups, which confer superior antioxidant properties. Research has shown that the dietary intake of ACNs not only eliminates free radicals but also reduces ROS levels induced by UV irradiation. Furthermore, it enhances the activity of antioxidant enzymes in vivo and reduces MDA content, thereby mitigating oxidative damage and potentially offering a protective effect against skin aging.

Cellular studies have consistently shown that ACNs possess significant antioxidant properties. *Vaccinium myrtillus* extracts enriched with ACNs have been shown to mitigate UVA-induced lipid peroxidation (LPO) and MDA generation, ultimately leading to a reduction in the apoptosis of HaCaT cells [46]. Lyu et al. [47] compared the photoprotective potentials of two ACNs extracted from *Acanthopanax divaricatus* var. *albeofructus* (ADA) fruits: cyanidin 3-galactoside and cyanidin 3-lathyroside. The results indicated that both monomers of ACNs could decrease the levels of SOD and CAT in human dermal fibroblasts (HDF), with cyanidin 3-lathyroside exhibiting a more potent antioxidant effect than cyanidin 3-galactoside. This suggests that ACNs from ADA fruits can alleviate cellular oxidative damage by enhancing the antioxidant defense system. Furthermore, in vitro studies have revealed that Acai berry extract, which is rich in ACNs, can elevate GSH levels while simultaneously reducing MDA levels, thereby decreasing ROS production in fibroblasts [48].

ACNs have also shown strong antioxidant effects in animal and clinical studies. C3G has been identified as a crucial active monomer of ACNs. Research indicates that C3G can reduce skin oxidative damage by inhibiting GSH consumption in murine skin and lowering MDA and lipid peroxide production [49]. Liu et al. [50] showed that in comparison to unencapsulated C3G, C3G encapsulated within chitosan nanoparticles could effectively reduce UVB-induced lipid peroxidation and MDA levels in the dorsal skin of mice, while also enhancing systemic antioxidant capacity. In a study where red raspberry ethanol extract (RBE) was applied to the dorsal skin of mice subjected to UVB irradiation, an increase in SOD and CAT activity was observed to increase after five days. This suggests that the photoprotective effects of RBE are attributed to its ability to remove excessive ROS generated by UVB exposure while enhancing the organism’s overall antioxidant capacity [51]. Additionally, grape skin extract enriched with ACNs can inhibit ROS production through the activation of the UVB-induced Nrf_2_/HO-1 pathway in ICR mice, which upregulates Nrf2-mediated antioxidant enzyme HO-1 [52]. To evaluate the photoprotective and anti-aging properties of standardized red-orange (*Citrus sinensis* (L.) Osbeck) extracts, a clinical trial was conducted involving 110 Asian and Caucasian volunteers who were randomly assigned to receive either a placebo or ACNs-rich orange extract. The study demonstrated that these extracts effectively alleviated skin erythema induced by combined UVA and UVB irradiation by improving the overall skin antioxidant capacity and reducing UV-induced lipid peroxidation [53].

Overall, ACNs reduce peroxide production by enhancing antioxidant enzyme activity while diminishing oxidative stress through the activation of the Nrf2/HO-1 pathway, thereby exerting their antioxidant effects.

### 4.2. ACNs Alleviate Inflammatory Responses

Under normal physiological conditions, the transcription factor NF-κB remains inactive in the cytoplasm. However, exposure of the skin to UV radiation has been shown to trigger ROS production, which subsequently activates NF-κB signaling pathway. This activation initiates IκB kinase activity, an enzyme responsible for phosphorylating inhibitors of NF-κB (IκB) proteins. Once phosphorylated IκB undergoes ubiquitination followed by degradation, this allows NF-κB translocation from the cytoplasm into the nucleus [54]. Within the nucleus, nitric oxide synthase is activated by NF-κB, leading to lipid peroxidation events that damage the cell membrane while promoting the secretion of inflammatory mediators. These biochemical processes collectively enhance the seven synthesis levels of various inflammatory mediators [55,56]. Pro-inflammatory cytokines enhance capillary permeability, leading to the exudation of white blood cells from blood vessel walls and the activation of inflammatory cells such as neutrophils and macrophages. This process triggers acute inflammation and contributes to skin photoaging [57].

ACNs can inhibit the release of inflammatory mediators, modulate the levels of pro- and anti-inflammatory cytokine levels, and alleviate both UV-induced cell damage as well as pathological changes in mouse skin. In vitro cell tests have demonstrated that C3G effectively reduces COX-2 levels in HaCaT cells by inhibiting the activation of mitogen-activated protein kinases (MAPKs) and PI3K/Akt pathways to alleviate inflammation [58]. Watcharaporn et al. [59] identified high concentrations of three specific ACNs (pelargonidin-3-glucoside, C3G, and peonidin-3-glucoside) in purple cornsilk extract. When applied to HaCaT cells, this extract inhibits the nuclear translocation and the expression of NF-κB, resulting in decreased NF-κB activity. Furthermore, treatment with ACNs-rich purple cornsilk extract significantly reduced UVB-induced pro-inflammatory cytokines, iNOS, and COX-2 levels compared to the UVB control group. During animal experiments conducted by Divya et al. [60], it was observed that the skin production of IL-6, TNF-α, iNOS, COX-2, and PGE2 in SKH-1 hairless mice subjected to UVB irradiation may be decreased through the application of ACNs-rich blackberry extract (BBE). The anti-photoaging effects of BBE occur via blocking the nuclear translocation of NF-κB and preventing IκBα degradation in mouse skin, thereby alleviating chronic inflammatory responses induced by UVB radiation to some extent. The oral administration of ethanol-extracted *Vaccinium uliginosum* samples rich in malvidin-3-*O*-galactoside and delphinidin-3-*O*-glucoside to mice with UVB-induced skin photoaging resulted in a reduction in IL-6, IL-12, and TNF-α expression within hairless mouse skin tissue, thus demonstrating notable anti-inflammatory effects [61]. Interestingly, a separate study indicated that extracts from blackberry, strawberry, and blueberry did not inhibit AP-1 activation induced by either UVB or UVC radiation; however, blackberry extract inhibited NF-κB activation in mouse epidermal cells in a time-dependent manner due to its high concentration of cyanidin-3-rutinoside (C3R) [62]. Additionally, the C3G-containing moisturizing gel demonstrated a dose-dependent reduction in COX-2 and IL-6 levels when applied topically to the dorsal skin of mice over an approximate duration of eight weeks. This finding suggests that mitigating inflammatory components may alleviate UV-induced chronic light damage [63]. In conclusion, interventions utilizing ACNs on photoaged cells and animal models have established that ACNs can inhibit the release of inflammatory factors and suppress inflammatory responses through various mechanisms.

### 4.3. ACNs Improve Collagen Synthesis

ECM components include elastin, collagen, proteoglycan, glycosaminoglycan, etc. [64]. Hydroxyproline (Hyp) is a crucial component of promoting collagen regeneration and facilitating skin repair [65,66]. UV irradiation can induce intracellular ROS generation which further stimulates the MAPK signaling pathway. This process facilitates the formation of an AP-1 complex between c-Jun and c-Fos while encouraging MMP production, stimulating collagen breakdown within the ECM, and contributing to skin aging [67]. Additionally, exposure to UV light can inhibit transforming growth factor-β (TGF-β) signaling pathway activation. Excess ROS generated within cells as a result of UV exposure impedes TGF-β release while reducing its binding affinity for TGF-β receptor II, consequently inhibiting phosphorylation cascades involving downstream signaling proteins such as Smads that suppress collagen synthesis [68,69]. Therefore, exposure to UV radiation, a primary inducer of skin aging, can lead to reduced collagen levels along with disruption in both the structure and function of the ECM. These detrimental effects are mediated through the activation of the MAPK signaling pathway and interference with the TGF-β signaling pathway.

ACNs play a crucial role in promoting collagen gene expression and synthesis, particularly type I collagen. The increase in ECM collagen content is associated with decreased degradation mediated by MMPs. Concurrently, ACNs effectively activate targets within the TGF-β/Smad signaling pathway, which promotes type I collagen synthesis while inducing the downregulation of MMPs. Tomasello et al. [70] demonstrated that extracts from three red-orange varieties rich in ACNs could decrease MMP levels and mRNA expression in human foreskin fibroblasts induced by UVB exposure, thereby mitigating ECM degradation induced by UVB exposure. An in vitro study on HaCaT and HDF cells revealed that black rice extract could repair UVB-induced structural alterations in the ECM by inhibiting AP-1 activity, reducing MMP release, and enhancing type I collagen expression [71]. Additionally, rose extract abundant in ACNs was shown to activate AP-1 while inhibiting MMP-1 levels, thus reducing collagen degradation [72]. Another investigation revealed that *Ribes nigrum* L. (RN) extract rich in C3G and C3R can enhance Smad2/3 phosphorylation along with TGF-β expression; this ultimately leads to increased procollagen and elastin synthesis. Furthermore, RN extract has been shown to reduce ERK and JNK phosphorylation, inhibit MMP-1 levels, and prevent collagen degradation, thereby providing photoprotection for HDF cells [73]. Additionally, in UVB-damaged HaCaT cells, fermented blackberry extract (FBB) containing ACNs reduced the levels of MMP-1 and MMP-2 proteins. Because FBB has a higher concentration of flavonoids and polyphenols than non-FBB, this results in an inhibitory effect on MMP-1 production [74]. In an animal study involving Wistar rats, a cream composed of 35% black rice bran extract was applied to the dorsal skin for four weeks following UVB irradiation. The results indicated that this cream inhibited MMP-1 levels in the skin while stimulating collagen synthesis within the dermis [75]. ACNs derived from purple sweet potato reduce AP-1 production and MMP-1 secretion by inhibiting MAPK signaling pathway activation. This results in decreased collagen breakdown along with increased Hyp content and water retention within mouse skin, ultimately improving skin hydration [76]. In summary, ACNs demonstrate a capacity to inhibit MMP secretion, promote type I procollagen synthesis, and maintain a balance between collagen synthesis and degradation.

### 4.4. ACNs Alleviate DNA Damage

DNA damage induced by UVR is closely linked to the formation of photoproducts. UVB, rather than UVA, serves as the primary contributor to erythema associated with sun exposure. UVB can directly inflict DNA damage and is approximately 1000 times more effective than UVA in causing sunburn [77]. While UVA induces oxidative DNA damage through the generation of excessive ROS, UVB directly facilitates the formation of 6-4 pyrimidine dimers and cyclobutane pyrimidine dimers (CPDs) via the absorption of UV energy [78]. This mechanism can disrupt DNA structure, impede DNA replication, induce skin photodamage, and accelerate skin aging [79,80]. The occurrence of DNA damage triggers G1 cell cycle arrest through the activation of p53 and telangiectatic ataxia mutant proteins, which subsequently initiate DNA repair mechanisms [81]. In cases where DNA sustains irreversible damage, it can activate the p53 signaling pathway leading to an upregulation in the expression of B-cell lymphoma-2-related X protein (Bax), Noxa, p53 levels in mitochondria, apoptosis regulatory factor proteins, and death receptor Fas, ultimately inducing apoptosis [82,83]. Prolonged exposure to UVR may result in p53 mutations that compromise its ability to regulate both cell death and DNA repair processes. Consequently, this allows damaged DNA to progress unchecked into mitosis, a potential precursor for skin cancer development.

Cellular and animal experiments have revealed that ACNs exhibit robust DNA protective activity and repair properties by reducing DNA double-strand breaks and that they exhibit apoptosis while promoting DNA repair processes. A study reported that C3G, whether administered alone or encapsulated in chitosan nanoparticles, can prevent skin cell apoptosis by downregulating p53 and Bax levels while upregulating Bcl-2 levels [50]. Additionally, research has indicated that C3G has a protective effect on HDF cells by reducing apoptosis through autophagy induction and decreasing oxidative stress caused by UVA exposure [84]. In vitro studies on HaCaT cells showed that C3G could reduce DNA damage, P53 level, mitochondrial membrane potential, and apoptosis induced by UVB radiation [85]. Bae et al. [86] reported that an ACNs-rich extract from bog blueberry could inhibit the UVB-induced apoptosis of HDF cells by decreasing ROS production while preventing DNA damage-mediated p53 signaling pathway activation. Blackberry juice, rich primarily in ellagitannins and ACNs, reduced CPDs and 8-oxo-7, 8-dihydro-20-deoxyguanosine (8-OhdG) levels while elevating caspase-1, caspase-8, and caspase-9 levels; this led to the enhanced apoptosis of damaged cells [87]. Furthermore, *Hibiscus syriacus* L., which is also rich in ACNs, concentrations of ACNs greater than 100 mg/L exhibited inhibitory effects on the caspase pathway while reducing activities of caspase-3 and caspase-7, consequently diminishing photodamage effects induced by UVB on HaCaT cells [88]. Another study confirmed that ACNs-rich black bean seed coat extract could prevent UVB-induced apoptosis in HaCaT cells by inhibiting caspase-3 pathway activation and down-regulating Bax protein levels [89]. The black soybean seed coat extract containing 72% C3G, 20% delphinidin-3-glucoside, and 6% petunian-3-glucoside was administered topically to the dorsal skin of hairless mice; it effectively inhibited UVB-induced apoptosis in mouse skin cells [89]. The local application of C3G on SKH-1 hairless mice’s dorsal skin effectively reduced 8-OhdG formation resulting from UVB exposure [49]. Additionally, the intragastric administration of ACNs extracted from black peanuts in mice led to the decreased production of 8-OhdG, suppression of P53 expression, inhibition of apoptosis, as well as mitigation of UVB-induced DNA damage [90]. Overall, these findings indicate that ACNs mitigate DNA damage by inhibiting markers associated with such damage while also reducing apoptosis through the inhibition of caspase-3- and P53-mediated signaling pathways.

### 4.5. ACNs Inhibit Pigmentation

Skin color is primarily determined by melanin, a pigment composed of amino acids produced by melanocytes located in the basal layer of the epidermis. When keratinocytes are exposed to UV light, particularly UVB radiation, there is an upregulation of pro-opiomelanocortin (POMC), which results in increased levels of the α-melanocyte-stimulating hormone (α-MSH) within these cells. α-MSH, a post-translational cleavage product of POMC, stimulates the melanocortin-1 receptor on adjacent melanocytes. This interaction activates microphthalmia-associated transcription factor (MITF), leading to subsequent melanin production [91,92]. Melanin is then transferred from melanocytes to neighboring keratinocytes via dendrites, resulting in skin pigmentation [93]. This complex process highlights the intricate mechanisms involved in melanin synthesis and its regulation of skin pigmentation.

The application of ACNs as agents for skin whitening to reduce pigmentation has also been explored as a common approach to alleviate photoaging effects on the skin [94,95,96]. Karunarathne et al. [88] demonstrated that ACNs derived from *Hibiscus syriacus* L. can inhibit melanin synthesis by reducing α-MSH stimulation and exert an anti-melanin effect through the downregulation of genes such as tyrosinase and MITF expression. Additionally, it has been discovered that fermented black rice inhibits melanin production in B16F10 cells. This inhibition may be attributed to decreased expression levels of tyrosinase and related proteins [97]. Furthermore, research involving rose extract rich in ACNs has indicated its efficacy in inhibiting tyrosinase activity while reducing melanin formation without causing cellular harm. A clinical study revealed that the topical application of rose extract on human skin enhances brightness, contributing to a whitening effect [72]. Another clinical trial indicated that the oral administration of ACNs-rich Macchi berry extract (MBE) capsules could improve skin brightness and alleviate erythema, suggesting that MBE possesses certain anti-photoaging properties for the skin [98]. Collectively, these findings imply that ACNs indeed have the potential to decrease pigmentation.

## 5. Application Forms of ACNs

When utilizing ACNs, it is essential to consider multiple factors to ensure their effectiveness given their low bioavailability and permeability. Beyond the inherent characteristics of ACNs themselves, one should also evaluate how different application forms, such as cream, liposomes, and moisturizing gels for topical applications, as well as encapsulation techniques and specific macromolecules for oral administration, impact efficacy.

The therapeutic benefits of the topical application of ACNs depend on their ability to penetrate the epidermis and reach subcutaneous layers. Various encapsulation methods for ACNs such as creams, liposomes, and moisturizing gels enhance their therapeutic efficacy when applied topically [99]. For instance, a pomegranate cream enriched with ACNs demonstrated notable anti-aging effects on volunteers’ skin along with an impressive rate of skin permeability [100]. In another study involving ACNs from blueberries encapsulated in liposomes, it was shown that they could effectively penetrate through the stratum corneum (SC) to reach the epidermis; this enhanced the photoprotective effects of ACNs on the skin [101]. Research has also indicated that lipophilic lipsticks formulated with ACNs from elderberry and red radish can overcome the SC barrier for effective permeation into the skin, with elderberry-derived ACNs exhibiting faster release due to their smaller molecular weight [102]. Lee et al. [103] demonstrated that hydrogenated soybean phosphatidylcholine (HSPC) forms a stable phospholipid bilayer capable of effectively encapsulating ACNs. Remote loading anthocyanin HSPC (RAH) liposomes improved both the loading efficiency and stability of ACNs compared to non-formulated anthocyanin (NFA) while exhibiting superior antioxidative capabilities relative to NFA and passive loading anthocyanin HSPC (PAH) liposomes. Additionally, C3G was found to exhibit favorable skin permeability when incorporated into moisturizing gel formulations [63].

In addition, the mechanisms underlying the digestion and absorption of ACNs following oral administration remain inadequately understood. This presents a challenge in elucidating their paradoxical high biological activities coupled with low bioavailability [104,105,106]. Research has shown that the primary site for ACN absorption is the small intestine, where they are decomposed into low-hydrophilic aglycones. This transformation facilitates their penetration through the phospholipid bilayer membrane with the assistance of carriers or via passive transport [107]. Han et al. reported that C3G undergoes hydrolysis within the gastrointestinal tract, with absorption predominantly occurring in the jejunum and ileum sections of the small intestine [108]. It has also been observed that ACNs are initially absorbed in the stomach post-ingestion [109]; however, due to the harsh pH environment present within the gastrointestinal tract, they become unstable and struggle to reach the intestine sites for further absorption [110]. These findings clarify why ACNs demonstrate high bioactivity yet exhibit low bioavailability following oral administration; thus, there is an urgent need to develop application methods capable of physically protecting ACN compounds while delaying their release and delivery within biological systems. Encapsulation emerges as an effective strategy to prevent ACN degradation. When alongside certain macromolecules or through encapsulation techniques, these compounds can effectively fulfill their intended roles; similar strategies are commonly employed in both pharmaceutical applications and functional food development.

The encapsulation types of ACNs, including nanoparticle, microencapsulation, emulsion, liposome, and hydrogel, are summarized in Table 1. The selection of encapsulation materials is a critical factor, as it influences both the encapsulation efficiency and stability of ACNs. Chitosan, pectin, soy protein isolate, and other matrix materials can be used as matrix materials for encapsulation. Materials such as chitosan, pectin, soy protein isolate, and other matrix substances can be employed for this purpose. Chitosan is particularly favored as a packaging material for bioactive compounds due to its favorable biocompatibility and biodegradability [111]. Consequently, chitosan is frequently utilized as a coating material in the formulation of ACN liposomes and nanoparticles. In vitro simulated gastrointestinal digestion experiments have demonstrated that incorporating chitosan into ACN liposomes can delay the release of ACNs while prolonging their retention time within the body [112]. Research has demonstrated that chitosan nanoparticles loaded with ACNs exhibit a slow-release effect and enhanced stability when compared to free ACN solution in simulated gastroenteric fluid [113]. Additionally, pectin serves as an abundant source of polysaccharides; when used as a wall-sealing material, it renders encapsulated ACNs insoluble in water, thereby preventing their degradation during gastric digestion, and allows them to exert effects at specific sites within the gastrointestinal tract [114,115]. Soybean protein isolate offers several advantages: it is resource-abundant, non-toxic, cost-effective, and possesses good biocompatibility along with biodegradability. This makes it an emerging functional food wall-sealing material with significant application potential [116]. The encapsulation of ACNs enhances their bioavailability and stability within the gastrointestinal tract, and these coating materials address issues related to the low utilization rates and poor stability associated with free-form ACNs [117].

The researchers also discovered that certain biological macromolecules such as some proteins, polysaccharides, and lipids can enhance the stability or bioavailability of ACNs (Table 2). In vitro simulated gastrointestinal digestion experiments have shown that α-casein and β-casein can improve the stability of ACNs during digestion, thereby enhancing their bioavailability. This indicates that these macromolecules play a dual role in both protecting ACNs and improving their bioavailability [129]. Additionally, the researchers evaluated the protective effect of quinoa octenyl succinate starch on ACNs during in vitro gastrointestinal digestion. They found that this starch could protect ACNs from gastric degradation by regulating their release through starch hydrolysis [130]. Furthermore, a human trial revealed that consuming ACNs-rich strawberries with cream resulted in an increased duration for stomach emptying and mouth reaching the cecum [131]. These encapsulation techniques and biological macromolecules play a key role in maximizing the bioavailability of ACNs while improving targeting accuracy.

## 6. The Potential of ACNs in Developing Functional Foods

ACNs hold significant promise as functional food ingredients due to their potent properties. Currently, they are incorporated into various functional foods such as bread, biscuits, beverages, etc. For instance, blue, pink, and purple corn kernels could be utilized to produce natural purple tortillas, demonstrating the practical applications of ACNs in food [142].

ACNs have demonstrated the ability to maintain antioxidant capacity in vitro, positioning them as a functional food. The researchers evaluated the antioxidant potential of whole wheat sticks, biscuits, bread, pancakes, and porridge enriched with black rice bran by assessing their efficacy in scavenging ABTS, DPPH, and peroxide free radicals in vitro. Their findings revealed that whole wheat bars and biscuits, which contain high levels of ACNs and dietary fiber derived from black rice bran, exhibited the most potent radical-scavenging abilities [143]. A study was conducted to verify whether elderberry juice containing compounds such as C3G and cyanidin-3-*O*-sambubioside retained its strong antioxidant activity when incorporated into croissants. The researchers assessed DPPH free radical scavenging, reducing power, and β-carotene bleaching in selected croissants. Although the results revealed that the antioxidant activity was lower than anticipated, it still demonstrated significant effectiveness. This may be attributed to the high temperatures involved in the baking process. Nonetheless, incorporating Sambubio juice offers beneficial effects [144].

Previous in vivo studies have demonstrated that functional foods rich in ACNs can effectively enhance cognitive function, reduce glycemic response, and protect against DNA damage. The administration of blueberry ACNs improved spatial working memory in elderly Wistar rats and increased levels of brain-derived neurotrophic factor in the hippocampus, indicating a potential benefit for age-related cognitive decline [145]. Moreover, a study involving elderly individuals experiencing memory loss but without dementia revealed significant improvements in language learning abilities after 12 weeks of consuming ACNs-rich grape juice supplements [146]. An intervention study conducted with children aged 7 to 10 years involved administering either a placebo or a blueberry drink containing 15 or 30 g of freeze-dried wild blueberry powder. The children underwent three cognitive assessments over a six-hour period, which indicated enhancements in verbal memory, word recognition, response interference, response inhibition, and processing speed [147]. In another human experiment, sixteen healthy subjects were recruited to provide blood samples during fasting and at various intervals post-consumption of different food items: 50 g each of wheat bread, riceberry rice bread, and Hom Mali bread, alongside Glucose as the reference, and then the blood glucose concentration in serum was measured within 30 to 180 min. Findings indicated that riceberry rice bread enriched with ACNs could lower blood glucose concentration among healthy individuals while improving plasma iron reduction capacity and enhancing overall antioxidant capacity [148]. Additionally, a clinical trial involving ten healthy male participants who consumed ACNs-rich blueberry for one hour showed an impressive reduction by 18% in H_2_O_2_-induced DNA damage in blood monocytes compared to a control group consuming jelly [149]. In summary, ACNs are increasingly being incorporated into food and beverage products as natural colorants while also demonstrating considerable potential for application in the development of functional foods and dietary supplements; this further amplifies their beneficial effects on human health.

## 7. Conclusions and Perspectives

**Figure 4:**
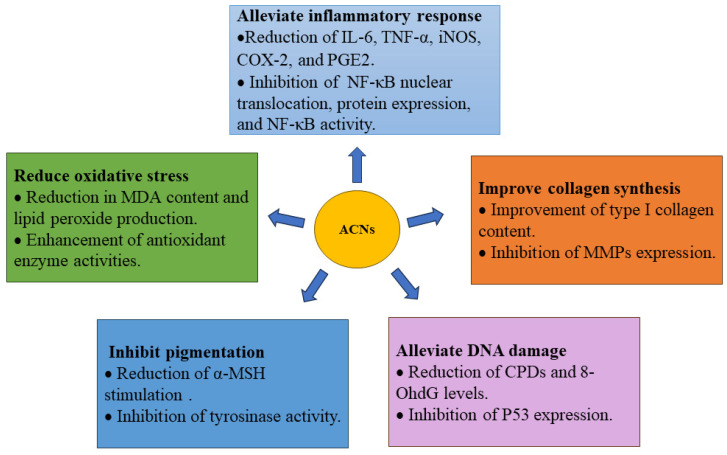
The beneficial effects of anthocyanins (ACNs) in repairing photoaged skin. ACNs, anthocyanins; NF-κB, nuclear factor-κB; IL-6, interleukin-6; iNOS, inducible nitric oxide synthase; COX-2, cyclooxygenase-2; TNF-α, tumor necrosis factor-α; PGE2, prostaglandin E2; MMPs, matrix metalloproteinases; CPDs, cyclobutane pyrimidine dimers; 8-OHdG, 8-oxo-7, 8-dihydro-20-deoxyguanosine; α-MSH, α-melanocyte-stimulating hormone; MDA, malondialdehyde.

ACNs are promising and effective candidates for the repair of photoaged skin. This review has highlighted various sources of ACNs found in fruits, vegetables, cereals, and flowers. Furthermore, we explored the potential of ACNs in combating skin photoaging by reducing oxidative stress, alleviating inflammatory responses, improving collagen synthesis, alleviating DNA damage, and inhibiting pigmentation (Figure 4). It discussed strategies to improve the stability and bioavailability of ACNs through methods such as microencapsulation, emulsions, liposomes, hydrogels, as well as utilizing proteins, polysaccharides, and lipids. The review has also addressed the potential applications of ACNs in developing functional foods and nutritional supplements while emphasizing their beneficial contributions to health and well-being.

Despite the complex structure of ACNs posing challenges for extraction and purification processes, future research should prioritize the development of more efficient extraction techniques to obtain a broader range of high-purity ACNs suitable for medical applications and skincare products. Furthermore, it is essential to investigate the synergistic effects between ACNs and other anti-photoaging agents while optimizing administration methods and dosages within clinical settings to establish effective treatment regimens for photoaged skin. In addition, further studies are necessary to elucidate the repair mechanisms of ACNs on skin photoaging at the cellular, animal, and clinical levels. While ACNs exhibit resistance to skin photodamage, their stability can be influenced by fluctuations in temperature and pH levels. Encapsulating ACNs with biomacromolecules significantly enhances both their stability and bioavailability. Future research could focus on developing more effective encapsulation methods to further improve these properties. Moreover, dietary supplements rich in ACNs—such as oral solutions or meal replacement powders—are anticipated to provide innovative approaches for addressing concerns related to skin photoaging. These initiatives will advance practical applications of ACNs in preventing and treating skin photoaging while enhancing our understanding of how photoprotective skincare products or functional foods can effectively mitigate this condition.

## Figures and Tables

**Table 1:** Encapsulation types of anthocyanins (ACNs).

Encapsulation Type	Coating Materials	Effects	Reference
Nanoparticle	β-cyclodextrin	Improved bioavailability and stability of ACNs (in vitro).	[118]
	gelatin, chitosan	Improved stability of ACNs (in vitro).	[119]
Microencapsulation	β cyclodextrin, β glucan	Delayed the release and improved stability of ACNs (in vitro).	[120]
	soy protein isolate, gelatin, maltodextrin, Arabic gum	Delayed the release of ACNs in the gastrointestinal tract (soy protein isolate and gelatin); showed no obvious influence on the release time of ACNs (maltodextrin, Arabic gum) (in vitro).	[121]
Emulsion	pea protein isolate, xanthan gum	Improved bioavailability and stability of ACNs (in vitro).	[122]
	gelatin Arabic gum, chitosan carboxymethyl cellulose	Improved the stability and antioxidant activity, and prolonged the half-life of ACNs (in vitro).	[123]
Liposome	soybean phosphatidylcholine	Improved the bioavailability of ACNs (in vitro).	[124]
	lecithin, chitosan	Improved the bioaccessibility of ACNs (in vitro).	[125]
Hydrogel	sodium alginate	Delayed the release of ACNs in the gastrointestinal tract and improved the thermal stability of ACNs (in vitro).	[126]
	κ-Carrageenan	Increased the retention time of ACNs in the stomach and improved their bioavailability (in vitro).	[127]
	alginate, pectin	Improved the encapsulation efficiency and stability of ACNs (in vitro).	[128]

**Table 2:** The effects of biological macromolecules on the bioavailability of anthocyanins (ACNs).

Combined Substances		Sources of ACNs	Effects	Reference
Protein	pea protein, rice protein	blueberry, grape	Improved the stability, antioxidant, and anti-inflammatory activity of ACNs (in vitro).	[132]
	whey protein	sour cherries	Prolonged the release of ACNs in the intestine (in vitro).	[133]
	whey protein	bilberry	Improved the stability and intestinal accessibility of ACNs (in vivo).	[134]
	β-lactoglobulin	grape skin	Reduced the degradation of ACNs and improved the stability of ACNs (in vitro).	[135]
	micellar casein, whey protein	blueberry	Delayed the release of ACNs in the gastrointestinal tract (in vitro).	[136]
Polysaccharide	pectin	blueberry	Improved the stability of ACNs in gastrointestinal tract simulation (in vitro).	[137]
	chitin-ethyl cellulose	grape seed	Reduced the release of ACNs in the gastrointestinal tract and promoted the release of ACNs in the colon (in vitro).	[138]
	acetylated distarch phosphate	red cabbage	Improved the stability of ACNs (in vitro).	[139]
Lipid	phospholipids	black rice	Improved bioavailability of phospholipids-black rice ACNs complex (in vivo).	[140]
	phospholipids	purple sweet potato	Improved the stability and antioxidant activity of ACNs, and promoted the ability of ACNs to be transported in the intestine (in vitro).	[141]

## Data Availability

The original contributions presented in this study are included in the article. Further inquiries can be directed to the corresponding authors.
